# Network Pharmacology Analysis and Experimental Validation of Tectoridin in the Treatment of Ischemic Stroke by Inhibiting Apoptosis and Regulating Inflammation

**DOI:** 10.3390/ijms26041402

**Published:** 2025-02-07

**Authors:** Tingting Lu, Zhen Feng, Huiming Xue, Chang Jin, Yue Zhang, Yongxing Ai, Meizhu Zheng, Dongfang Shi, Kai Song

**Affiliations:** 1 College of Life Sciences, Changchun Normal University, Changchun 130032, China; lutingting2580@163.com (T.L.); fengzhen0915@163.com (Z.F.); 13844637912@163.com (H.X.); j1504408478@163.com (C.J.); 15942907284@163.com (Y.Z.); songkai@ccsfu.edu.cn (K.S.); 2 College of Animal Science, Jilin University, Changchun 130062, China; aiyx@jlu.edu.cn; 3 Central Laboratory, Changchun Normal University, Changchun 130032, China

**Keywords:** network analysis, tectoridin, ischemic stroke, animal experiments, neuroprotection

## Abstract

The flowers of *Pueraria lobate* (Puerariae Flos) have served as a traditional Chinese medicinal and food herbage plant for many years. Tectoridin is one of the most active metabolites extracted from flowers of *Pueraria lobate* and has a variety of beneficial activities, including antioxidative, hypoglycemic, and anti-inflammatory activities. Nevertheless, the functions and potential mechanisms underlying tectoridin in cerebral ischemia/reperfusion injury have not been well interpreted; thus, a network analysis strategy was performed to systematically evaluate its pharmacological mechanisms, which were further validated in rats with cerebral ischemia. Network analysis predicted that tectoridin could attenuate brain damage after stroke by modulating signaling pathways associated with redox, inflammation, and autophagy. The experimental results demonstrated an improvement in neurological function in rats treated with tectoridin, along with a significant reduction in cerebral infarction volume. The neuroprotective benefits of tectoridin stem, in part, from its antioxidant capabilities, which include the upregulation of Nrf2/HO-1 protein expression, reduction of the TLR4/MYD88/NF-κB inflammatory pathway, and inhibition of the PI3K/Akt/mTOR pathway, contributing to its anti-apoptotic effects. This investigation offers a thorough examination of the pathways and targets linked to the therapeutic effects of tectoridin on ischemic stroke, highlighting its anti-inflammatory, antioxidative, and anti-apoptotic mechanisms. These findings serve as a valuable reference for the development and exploration of effective anti-ischemic stroke medications.

## 1. Introduction

Stroke, as a type of disease with high prevalence, is the second leading cause of death worldwide and the critical cause of acquired disability in adults [1]. Stroke can be divided into ischemic stroke (IS) and hemorrhagic stroke, and up to 87% of stroke cases in the world are attributed to IS [2]. Thrombolysis is the only useful treatment to date, though it can only be used to treat 5% of stroke patients [3]. This fact leads to the search for a chemical capable of preventing neurons from undergoing stroke damage through the interruption of the biochemical cascade that results in cell death in the penumbra. Though an increasing amount of research has focused on stroke [4], few effective therapeutic drugs have been applied clinically. Currently, four types of acetylcholinesterase inhibitors (donepezil, galantamine, rivastigmine, and tacrine) are used to treat stroke via the agonist (memantine) of one N-methyl-D-aspartate (NMDA) receptor [5]. Nevertheless, these drugs generate several side effects considering the nonselective effects on different central and peripheral tissues and organs [6]. Accordingly, it is necessary to probe effective complementary and alternative therapies for stroke that can generate fewer side effects.

Cerebral ischemia-derived brain injury includes an inflammatory response, neuronal apoptosis, blood–brain barrier destruction, and other pathophysiological systems [7]. According to studies, excessive inflammation and immune response constitute the pathophysiological foundation of cerebral-infarction-induced ischemic brain injury [8]. Phosphatidylinositol 3-kinases (PI3Ks) together with their downstream target Akt are involved in a conserved signal transduction enzyme family and critically impact inflammatory response and apoptosis regulation [9]. The first few hours of cerebral ischemia are accompanied by the temporary elevation of p-Akt protein levels in neurons, which is considered one neuroprotective response [10]. P-Akt activates downstream proteins, like Bcl-2-associated X (Bax) and caspase.

As reported, nuclear factor erythroid-2-related factor 2 (Nrf2) exhibits a crucial relevance to antioxidant defense mechanisms and ROS scavenging under oxidative stress conditions [11]. In resting state, Nrf2 can be bound to Kelch-like ECH-associated protein 1 (Keap-1) and is maintained in an inhibitory state. The oxidative stress promotes the dissociation of Nrf2 from Keap-1 and its translocation to the nucleus, and in the nucleus, Nrf2 is bound to the antioxidant response elements (AREs) in promoter regions, acting as a battery for detoxifying genes and antioxidant genes, like heme-oxygenase-1 (HO-1) and superoxide dismutase (SOD) [12]. The therapeutic value exhibited by targeting the Nrf2/HO-1 pathway in cerebral ischemia–reperfusion (I/R) injury has been put forward in recent studies [13].

On the other hand, ROS can assist in simulating key signaling molecules (toll-like receptors (TLRs)), which remarkably regulate the inflammatory response, innate immunity, cell proliferation, and apoptosis [14,15]. TLR4 essentially constitutes TLRs and critically affects the pathogenesis regarding the brain, kidney, heart, and liver I/R injury [16,17,18]. Common signaling cascades regarding TLR4 are the MYD88-dependent and -independent pathways (MYD88, myeloid differentiation primary response gene 88) that stimulate nuclear factor-kappa B (NF-κB) and induce the release of pro-inflammatory cytokines and apoptosis [19,20].

In recent years, increasing attention has been paid to natural products with good therapeutic effects and few side effects [21]. Studies have found that natural products have a variety of biological activities, such as antifungal, antioxidant, antiviral, and anti-inflammatory, and have great potential for the development of new drugs, which can be used as a platform for discovering new targets and mechanisms of action and expanding chemical diversity [22,23,24]. Puerariae Flos, known as “Ge-hua” in Chinese, is botanically from the dried flowers of Pueraria montana var. thomsonii (Benth.). As a traditional Chinese medicine, it has been used to relieve toxic symptoms caused by excessive alcohol consumption, such as hangover, nausea, headache, and red face, in China, Japan, and Korea for over 1500 years [24]. The existence of isoflavones in Flos Puerariae has attracted the interest of scientists because they can confer health benefits upon the human body [25,26]. Tectoridin is a type of natural product exhibiting many pharmacological activities, including antioxidative [27], hypoglycemic [28], and anti-inflammatory activities [29], together with inhibitory activity against aldose reductase [30]. Nevertheless, the functions and mechanisms underlying tectoridin in cerebral I/R injury are still undiscovered.

Edaravone is an effective antioxidant capable of eliminating the free radicals that cause the development of many nervous system disorders. Animal studies together with clinical trials confirm its neuroprotective effect against acute cerebral infarction [31], intracerebral hemorrhage [32], subarachnoid hemorrhage [33], and other brain disorders [34]. Therefore, this study took edaravone as a positive control.

Network pharmacology merges disciplines, such as genomics, proteomics, and systems biology, to unveil connections among diseases, their targets, and pharmaceutical agents, facilitating a deeper comprehension of drug pharmacodynamics and their influence on biological networks [35,36,37]. Network analysis is an important part of network pharmacology and a research tool, which is capable of digging deeper into the nodes, edges, and sub-networks in the network to discover potential drug targets, mechanisms of action, etc.

**Figure 1:**
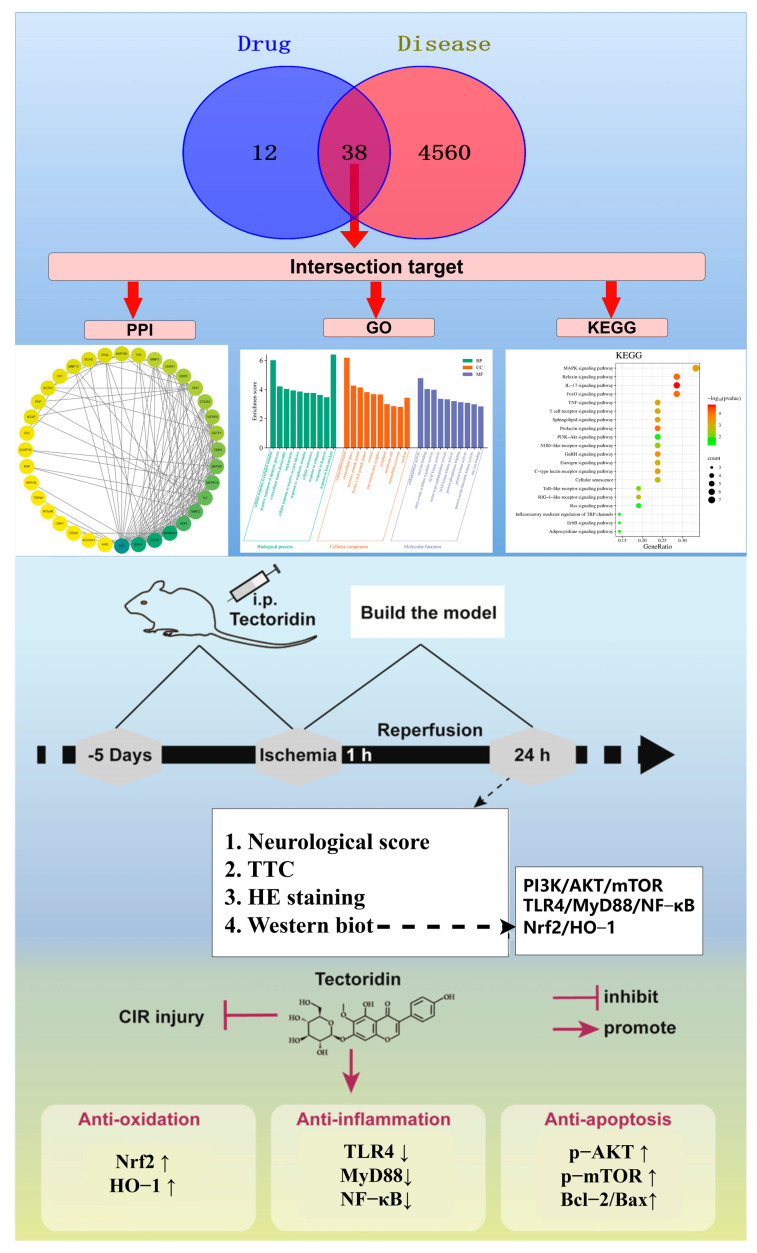
The experimental flow chart of the protective effect exerted by tectoridin in ischemic stroke. Note: “↑” indicates upregulation of protein expression, and “↓” indicates downregulation of protein expression.

Investigating the mechanism that tectoridin offers neuroprotection against cerebral injury, this research was designed to delineate the protective mechanisms of tectoridin in the context of ischemic stroke through the lens of network analysis. The methodology employed encompasses analyses of PPI networks, GO annotations, and KEGG pathway enrichment. Following these analytical processes, experimental validation was conducted utilizing an in vivo ischemic stroke model. The comprehensive results of this study shed light on the specific genes and pathways of tectoridin targets, contributing valuable insights and theoretical foundations for enhancing therapeutic strategies for ischemic stroke recovery. The complete research process is illustrated in Figure 1.

## 2. Results

### 2.1. Network Pharmacology Predicted the Biological Process and Enrichment Analysis of Tectoridin Against Ischemic Stroke

**Figure 2:**
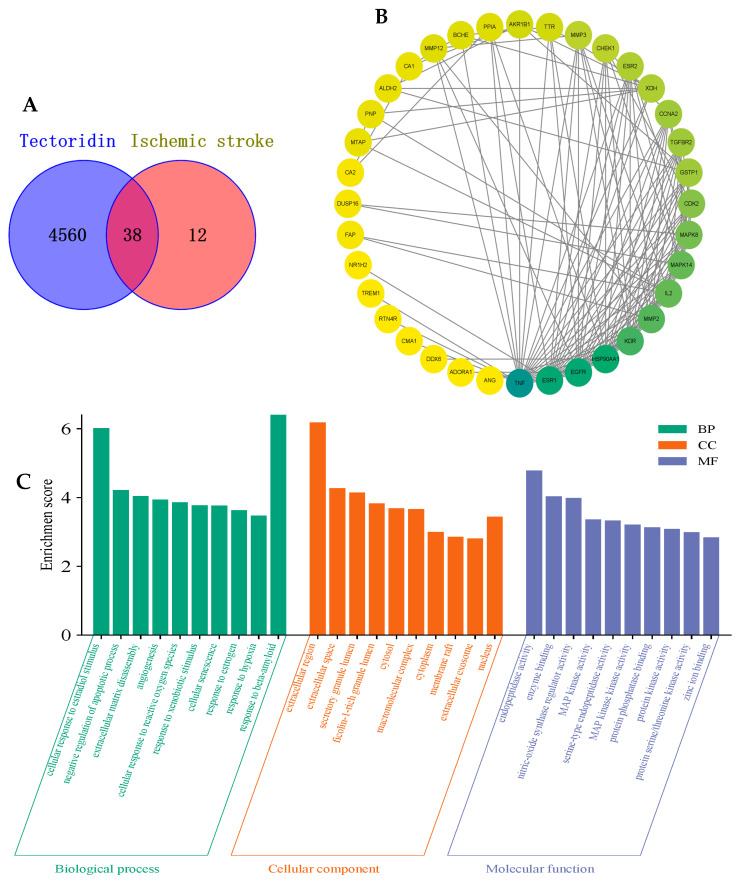

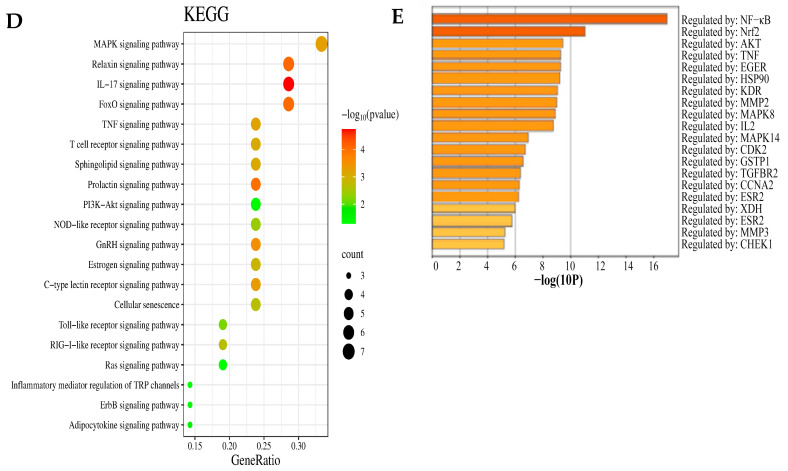
Protein–protein interaction (PPI) network and enrichment analysis of the anti-ischemic stroke mechanisms of tectoridin. (**A**) The 38 common targets of tectoridin and ischemic stroke. (**B**) PPI network plotting of main common targets. (**C**) GO enrichment analysis (the top 10 terms of BP, CC and MF enrichment analysis were shown in green, blue, and orange bars, respectively). (**D**) KEGG was significantly enriched in the top 20 pathways. (**E**) The protein enrichment analysis of tectoridin against ischemic stroke in TRRUST using Metascape (v3.5.20240901).

To explore the multiple targets and various pathways of tectoridin against ischemic stroke, we performed network pharmacology. First and foremost, we predicted the possible targets of tectoridin and obtained 101 potential related targets after eliminating the duplicates. Meanwhile, 4560 ischemic stroke-related targets were collected from the integration of Online Mendelian Inheritance in GeneCards, OMIM, PharmGkb, and DisGeNET databases. Finally, a total of 38 common protein targets of tectoridin and ischemic stroke were obtained (Figure 2A), which were subsequently input into the STRING 11.5 platform. Finally, a clearer protein–protein interaction (PPI) network diagram was depicted in Cytoscape software (version 3.8.0), including 38 main common nodes and 129 edges in total, with disconnected nodes being hidden (Figure 2B). At the same time, to reveal the functions of tectoridin in ischemic stroke treatment, we analyzed the biological process (BP), cellular component (CC), and molecular function (MF) in terms of the common targets with the Database for Annotation, Visualization and Integrated Discovery (DAVID). In total, 129 GO terms were significantly enriched (*p* < 0.05), including 80 BP terms, 17 CC terms, and 32 MF terms (Supplementary Table S1). The top 10 significantly enriched terms in the three categories are displayed visually (Figure 2C). The results showed that the main BP terms were extracellular matrix disassembly, apoptotic process, oxidative stress, response to xenobiotic stimulus; the main CC terms were extracellular region, secretory granule lumen, macromolecular complex, and cytoplasm; and the main MF terms were endopeptidase activity, enzyme binding, nitric-oxide synthase regulator activity, serine-type endopeptidase activity, MAP kinase kinase activity, and protein phosphatase binding.

Furthermore, we conducted a KEGG pathway enrichment analysis on 36 main common targets, and 59 pathways (*p* < 0.05) were identified (Supplementary Table S2). The top 20 signaling pathways were selected for visual display (Figure 2D). The results showed that the targets were enriched mainly in the PI3K-Akt signaling pathway, Toll-like receptor (TLR) signaling pathway, IL-17 signaling pathway, FoxO signaling pathway, MAPK signaling pathway, TNF signaling pathway, ErbB signaling pathway, modulation of TRP channels by inflammatory mediators, Ras signaling pathway, neurotrophin signaling pathway, and the process of cellular apoptosis. Most of the enriched signaling pathways were associated with the regulation of cell function, indicating that tectoridin may treat ischemic stroke by regulating the functions of various central and peripheral cells.

**Figure 3:**
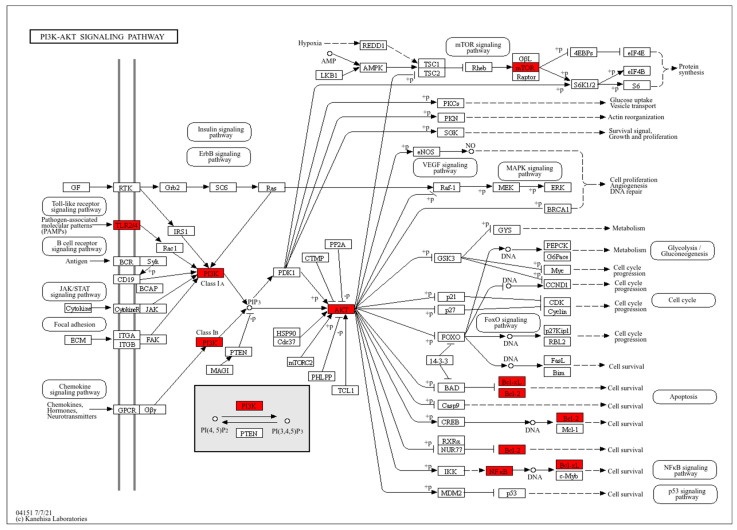
Core genes of key signaling pathway map of tectoridin against ischemic stroke. Note: The genes shown in red nodes are some of the key targets of tectoridin against ischemic stroke.

In addition, AKT and Nrf2 may be key contributors in the treatment of ischemic stroke with tectoridin (Figure 2E). In terms of key signaling pathways (Figure 3), based on analysis of the tectoridin-stroke PPI network, GO and the KEGG pathway enrichment analysis suggested that PI3K/Akt, Nrf2/HO-1, and TLR4/MyD88/NF-κB play an important role in the therapeutic actions of tectoridin for the treatment of stroke. Thus, network pharmacology analysis provided the direction for follow-up exploration of the mechanisms in play. Therefore, in the next study, we selected these three signaling pathways for experimental validation.

### 2.2. Tectoridin Down-Regulated Rats’ Neurological Impairment Scores and Infarct Volume After I/R

**Figure 4:**
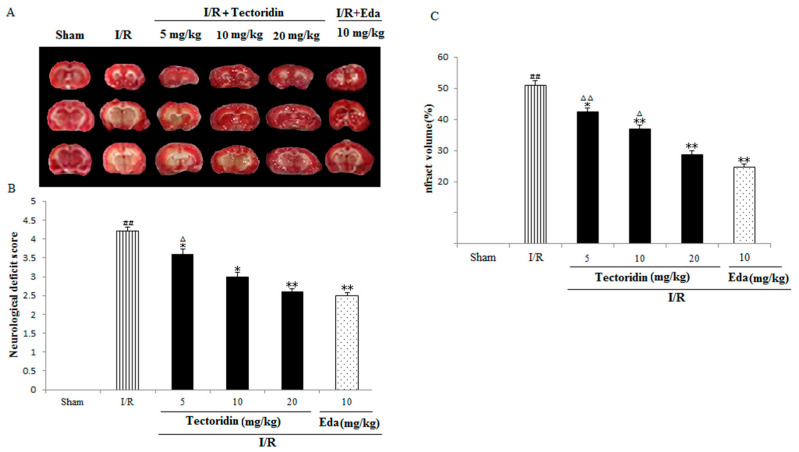
Tectoridin weakened the neurological deficits and infarct volume of ICR rat after I/R. (**A**) The impact of tectoridin on neurological deficits. (**B**) Representative brain sections after TTC staining. (**C**) The impact of tectoridin on infarct volume. ^##^ *p* < 0.01, compared with the Sham group; * *p* < 0.05, ** *p* < 0.01 vs. I/R. ^Δ^ *p* < 0.05, ^ΔΔ^ *p* < 0.01, relative to the Eda group.

Neurological impairment was tested using a 5-point scale upon the occurrence of ischemic stroke, and higher scores reflected more severe motor impairment. The results are presented in Figure 4A. Rats in the sham cohort exhibited no neurological impairment; hence, the cohort had a constant neurological score of zero across the entire study. Related neurological impairment happened inside the I/R cohort (irregular posture and weaker spontaneous activity). The mentioned indicators received the remarkable relieving process inside the tectoridin cohort at 5, 10, and 20 mg/kg (*p* < 0.05, *p* < 0.05, *p* < 0.01) and Edaravone at 10 mg/kg (*p* < 0.01).

As shown in Figure 4B,C, infarct volume presented an obvious increase in the ischemia group versus the sham group (*p* < 0.01). Infarct volume markedly decreased in the tectoridin group (5, 10, and 20 mg/kg) and Edaravone (10 mg/kg) group relative to the ischemia group, clearly proving the effect of Tec on alleviating the formation of brain edema after ischemic stroke (*p* < 0.05, *p* < 0.01, *p* < 0.01, *p* < 0.01) (Figure 4B,C).

### 2.3. Effect of Tectoridin on Pathological Brain Tissue Change

**Figure 5:**
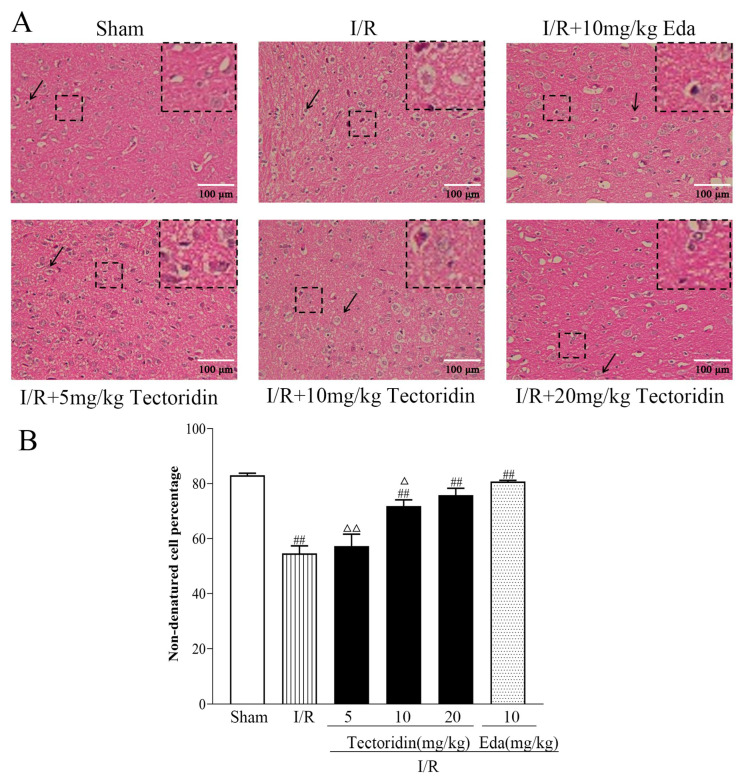
HE staining effect of tectoridin on the ischemic damage after I/R. (**A**) The morphology of nerne cells in the cortex of rats. (**B**) Non-denatured cell percentage bar chart. Values are in the form of means ± SEM (*n* = 3). ^##^ *p* < 0.01 obviously different from Sham; I/R; ^Δ^ *p* < 0.05, ^ΔΔ^ *p* < 0.01, relative to the Eda group.

HE staining showed that the sham cohort did not present abnormal brain tissue, with orderly arranged neurons, normal morphology, clear nucleolus, and uniform staining. There are rich cells in the hippocampus and abundant cell levels, and the cerebral cortex has rich cells without cell contraction. The corpus callosum has no cell edema or damage. In the ischemia cohort, the hippocampus and cerebral cortex show loose, edematous, and contracted cells. Some white matter cells have severe edema changes, larger ischemic area, and squeezed neurons, and the overall animal brain tissue is more serious. Nevertheless, pretreatment with 10 and 20 mg/kg tectoridin and 10 mg/kg Edaravone remarkably reduced the neuron pathological changes in the ischemic area. Meanwhile, the non-degeneration cell percentage in the I/R group was significantly lower than that in the sham group *(p* < 0.05). However, in the tectoridin (10 and 20 mg/kg) and Edaravone groups, the non-degeneration cell percentage was significantly increased versus the I/R group (*p* < 0.01, *p* < 0.01, *p* < 0.01). Accordingly, tectoridin improved nerve regeneration following the traumatic nerve injury (Figure 5).

### 2.4. Effect of Tectoridin on Nrf2/HO-1 Pathway

**Figure 6:**
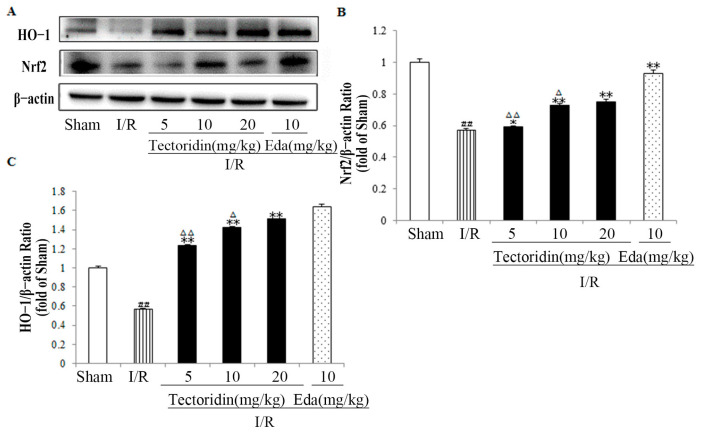
Effect of tectoridin on (Nrf2)/hemeoxygenase-1 (HO-1) pathway in the cerebral I/R injury of rats. (**A**) Nrf2 and HO-1 expressions. (**B**) Nrf2/β-actin ratio. (**C**) Ratio of HO-1/β-actin. Values are in the form of means ± SEM (*n* = 3). ^##^ *p* < 0.01 obviously different from Sham; * *p* < 0.05, ** *p* < 0.01, obviously different from I/R; ^Δ^ *p* < 0.05, ^ΔΔ^ *p* < 0.01, relative to the Eda group.

To examine how tectoridin affected Nrf2 and HO-1, this study further evaluated its protein levels in rat brain tissues. According to Figure 6, I/R remarkably decreased Nrf2 and HO-1 expressions relative to sham-operated control rats; comparatively, the I/R rats pretreated with tectoridin presented obviously increased Nrf2 and HO-1 levels relative to the I/R untreated rats.

### 2.5. Effect of Tectoridin on TLR4/MYD88/NF-κB Signaling Pathway

**Figure 7:**
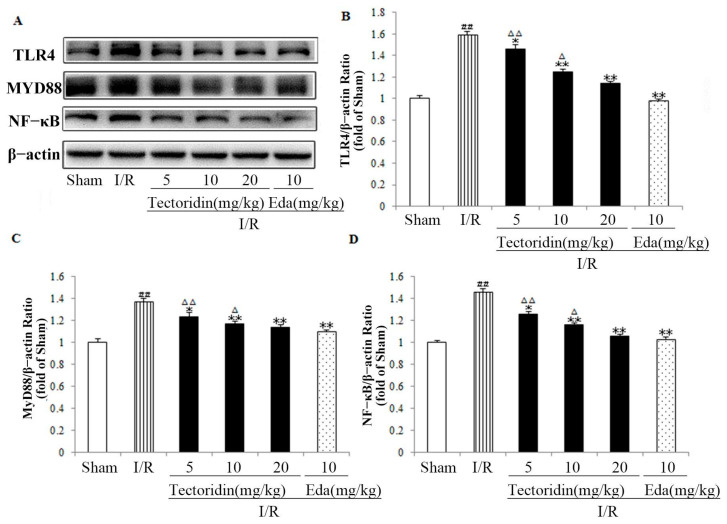
Tectoridin regulates the TLR4/MyD88/NF-κB pathway in the cerebral I/R injury of rats. Western blot (**A**) and analysis of TLR4 (**B**), MyD88 (**C**), NF-κB (**D**). Values are in the form of means ± SEM (*n* = 3). ^##^ *p* < 0.01 remarkably different from Sham; * *p* < 0.05, ** *p* < 0.01, remarkably different from I/R; ^Δ^ *p* < 0.05, ^ΔΔ^ *p* < 0.01, relative to the Eda group.

To evaluate the potential mechanism, the protein expression of the TLR4/MYD88/NF-κB pathway was detected. As expected, relative to the sham group, I/R stimulation led to obvious upregulated expression of TLR4, MyD88, nucleus NF-κB (*p* < 0.01, *p* < 0.01, *p* < 0.01). However, tectoridin incubation led to an obvious decrease in their expressions (*p* < 0.01, *p* < 0.01, *p* < 0.01), relative to the Edaravone group (*p* < 0.05) (Figure 7).

### 2.6. Tectoridin Down-Regulated the Expression of PI3K/Akt/mTOR Signaling Pathway in I/R Injury Rats

**Figure 8:**
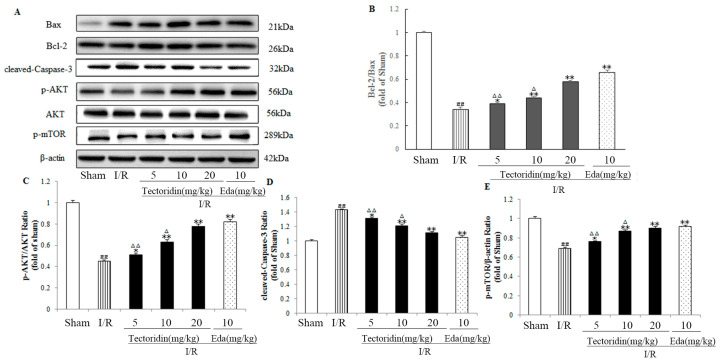
Effect of tectoridin on the PI3K/Akt signaling and the downstream proteins related to apoptosis. Western blot (**A**) and analysis of Bcl-2/Bax (**B**), p-Akt/Akt (**C**), cleaved Caspase-3 (**D**) and p-mTOR (**E**). Values are in the form of means ± SEM (*n* = 3). ^##^ *p* < 0.01 considerably different from Sham; * *p* < 0.05, ** *p* < 0.01, considerably different from I/R; ^Δ^ *p* < 0.05, ^ΔΔ^ *p* < 0.01, relative to the Eda group.

According to Figure 8, the I/R treatment group showed considerably lower proportions of p-mTOR and p-AKT relative to the control group (*p* < 0.01). Nevertheless, treatment with 10 and 20 mg/kg tectoridin remarkably inhibited this decrease (*p* < 0.01, *p* < 0.01), relative to the I/R group, which was considerably lower relative to the Edaravone group (*p* < 0.05).

The Western blot assay results in Figure 8 also showed that cerebral I/R significantly increased the levels of the cleaved-Caspase-3 and Bcl-2/Bax ratio. However, the administration of 20 mg/kg tectoridin observably reduced the expression level of cleaved-Caspase-3 and Bcl-2/Bax ratio, much lower relative to the Edaravone group (*p* < 0.01).

## 3. Discussion

The investigation into stroke treatment via tectoridin remains at an early stage of scientific inquiry. Our research pioneered a comprehensive exploration of the potential mechanisms behind tectoridin’s effect on stroke through the lens of network analysis, establishing the groundwork for future studies. In the realm of modern pharmacological research, network analysis has emerged as a pivotal technique for elucidating the intricate relationships between diseases, therapeutic agents, and the journey of drug development [38,39,40,41]. This study embarked on constructing a detailed metabolite–target interaction network, employing both network analysis and molecular docking strategies to unravel the complex molecular interactions of tectoridin in counteracting ischemic stroke (IS). The initial phase involved the identification of 101 tectoridin targets from established TCM and Swiss TargetPrediction databases, alongside IS-related targets derived from the TTD database. Subsequent alignment processes highlighted 38 intersecting targets between tectoridin’s predicted targets and those associated with IS. Analysis of the “tectoridin-target pathway” network demonstrated that tectoridin’s therapeutic influence on IS engages several critical pathways, including PI3K/AKT, Nrf2/HO-1, and TLR4/MYD88/NF-κB. This foundational research was further extended to confirm the central targets within these signaling pathways through rigorous molecular docking techniques and empirical in vivo experiments, guided by the insights from network analysis findings.

The PI3K/AKT signaling pathway, known for promoting cellular survival, plays a pivotal role in controlling cell growth, differentiation, metabolism, and resistance to cell death [42]. Activated during the initial phase of cerebral ischemia, this pathway contributes to the onset of apoptosis and inflammation. However, as ischemic conditions intensify, the pathway’s activity is progressively diminished [43]. According to various studies, AKT activation exerts a crucial effect on neuronal survival after the cerebral I/R injury [44]. Akt/mTOR signaling is involved in ischemic cardiomyocyte and apoptosis cardiovascular disease [45,46]. Docking of Gomisin N with PI3K, AKT and mTOR complexes was performed using Autodock Vina software 1.5.7 to determine the interaction between molecular dynamics simulations of PI3K, AKT or mTOR, and GN. Therefore, the PI3K/AKT/mTOR pathway is possibly involved in the protective effect of GN against brain I/R injury and its mechanism [47]. According to Western blotting analysis, the ischemic area presented improved p-mTOR expression. The PI3K/Akt pathway facilitated the neovascularization and led to obviously reduced infarct size following ischemia [48]. Nevertheless, research has confirmed different levels of phospho-Akt after the ischemic injury. Osuka and collaborators demonstrated that Akt pathway dysfunction was involved in ischemic damage [49], but Noshita and Shibata confirmed the transient increase in pAkt in neurons after the cerebral ischemia within hours [50,51], and such an increase confirmed the neuroprotective effect. Gao and Ishrat observed p-Akt up-regulation 24 h after I/R relative to the sham group [52,53]. Consistently, our study obtained the same result. According to other studies, total flavonoids of Chuju can inhibit HeLa cell proliferation and trigger apoptosis, during which process the PI3K/AKT/mTOR signaling pathway plays its role [54]. Studies have confirmed the key role played by the proteins caspase, Bcl-2, and BAX in the apoptotic process [55]. According to many studies, intrinsic or extrinsic pathways are capable of inducing apoptosis. The activation of caspase only occurs upon the cleavage of caspase and the activation of initiator caspases (caspase-3) [56]. The Bcl-2 family takes charge of balancing the up-regulation and down-regulation of proteins in terms of the mitochondrial potential of Bcl-xL, Bcl-2, and BAX (proapoptotic protein), which determine the cell apoptosis or survival [57]. Hence, this study will pay more attention to investigating the PI3K/AKT/mTOR pathway and cleaved caspase-3, BAX, and BCL-2, as well as further identifying the possible mechanisms [58].

The PI3K/AKT/mTOR signaling pathway possesses a lot of molecule subtypes, and each subtype contains a respective action mode and a lot of phosphorylation sites for controlling cells’ anti-apoptosis properties [59,60]. The apoptosis, neuronal injury, and cerebral infarct area were all evaluated, finding the involvement of PI3K in the neuroprotective of resveratrol on cerebral I/R. In addition, according to Western blot, the vehicle group after I/R presented reduced protein expressions of p-AKT and p-mTOR, which, however, were increased by resveratrol following one day of cerebral I/R (Figure 8). According to Western blot, BLC-2 was obviously higher and BAX and cleaved caspase-3 were obviously lower in the tectoridin group relative to the vehicle group (Figure 8). Hence, the PI3K/AKT/mTOR pathway greatly contributed to tectoridin’s neuroprotective and anti-apoptotic effects on cerebral I/R injury. Also, tectoridin down-regulated the BAX and cleaved caspase-3 proteins and up-regulated BCL-2 protein via activating the PI3K/AKT/m-TOR pathway, thereby inhibiting cell apoptosis.

In addition, the Nrf2/HO-1 signaling pathway is fundamentally involved in controlling the expression of genes critical for antioxidant defense and anti-inflammatory actions [61]. In the context of I/R injury, Nrf2 is translocated into the nucleus to activate HO-1, subsequently leading to a reduction in the expression of pro-inflammatory proteins [13]. This mechanism is crucial as Nrf2 activation enhances the transcription of antioxidant genes, thus playing a vital neuroprotective role during the onset and progression of ischemic stroke [62,63]. Our observations aligned with these findings, showing a decrease in the protein levels of Nrf2 and HO-1 after I/R injury. However, the administration of tectoridin significantly increased the expression levels of Nrf2 and HO-1, suggesting its potential therapeutic efficacy in ischemic stroke through modulation of the Nrf2/HO-1 pathway.

The TLR4/NF-κB signaling pathway serves as a fundamental pathway medicating the inflammation and remarkably impacts the ischemic injury of brains, hearts, and livers [64]. Without any stimulus, in the cytoplasm, NF-κB presents typical sequestering with IκB. Under stressful stimulation, through phosphorylation and deactivation of IκB kinase-β (IKKβ), IKBα releases NF-κB and translocates it to the nucleus as well as guiding many pro-inflammatory cytokines to transcript [65]. TLR4 assists in activating NF-κB via the MYD88-dependent pathway and MYD88-independent pathway, with the former stimulating the NF-κB to participate in the release of many inflammatory factors (TNF-α, interleukin-1 (IL-1), IL-6, and IL-8) [66]. For detecting whether tectoridin can resist inflammation via the TLR4/MYD88/NF-κB signaling pathway, this study focused on determining the expressions of TLR4, MYD88, and NF-κB at the protein level. As shown, the I/R group presented obviously increased protein expressions relative to the sham-operated rats, while tectoridin treatment led to obviously reduced expressions of the above indicators. The findings confirmed those of Niu et al., who revealed that tectoridin could modulate rats’ TLR4/NF-κB/NLRP3 signaling pathway to exert protective effects against the inflammatory response in inflammation induced by lipopolysaccharide [67,68]. Accordingly, tectoridin could inhibit the TLR4/MYD88/NF-κB signaling pathway, thereby decreasing the I/R-caused inflammatory response. Further experiments are needed to explore which aspect contributes more to the occurrence and development of cerebral I/R injury.

In conclusion, the mechanism of tectoridin in the treatment of cerebral ischemic stroke by inhibiting apoptosis and regulating neuroinflammation was investigated using network pharmacology analysis and experimental validation in vivo. The results demonstrated that tectoridin might reduce the number of apoptotic cells and regulate the expression of apoptosis-related proteins through the PI3K/AKT/mTOR signaling pathway, alleviating cerebral I/R injury by activating the antioxidant signaling pathways Nrf2/HO-1 and down-regulating the TLR4/MYD88/NF-κB inflammatory pathway. Tectoridin, as a natural drug, has the limitation of a slow therapeutic effect; however, it has the advantage of multi-target and multi-session overall regulation. Compared with the traditional Chinese medicine compound, tectoridin has clear active ingredients and better quality control; compared with synthetic drugs, tectoridin has less toxicity and fewer side effects. In conclusion, these results suggest that tectoridin is a promising drug for ischemic stroke treatment.

## 4. Materials and Methods

### 4.1. Network Pharmacology-Based Analysis

#### 4.1.1. Prediction of Tectoridin and Ischemic Stroke-Related Targets

The structure of tectoridin was obtained from the PubChem database [69], while its associated targets were predicted using the Swiss TargetPrediction [70] and PharmMapper databases [71]. The data from these sources were amalgamated and normalized using the Uniprot database to form a comprehensive list of tectoridin-related targets. GeneCards [72], OMIM [73], PharmGkb [74], and DisGeNET [75] databases were used.

#### 4.1.2. Creating a Protein Interaction Network (PPI) Network and the Core Gene Finding

PPI network is composed of proteins interacting with each other, which participate in various aspects of life processes, such as biological signaling, gene expression regulation, energy and material metabolism, and cell cycle regulation. The study of PPI network can help to explore the core regulatory genes. Among the various protein interaction databases, STRING is the one that covers the largest number of species and the largest amount of interaction information, which uses a spring model to generate network images, where the nodes are modeled as the masses and connections of a spring; the final positions of the nodes are calculated by minimizing the “energy” of the system.

The isolated intersecting target genes were integrated into the STRING database (version 12.0). Within this resource, “Homo sapiens” were selected as the specified species, and the interaction confidence level was set at a medium threshold of 0.4. After the integration, in order to perform a comprehensive topological analysis to highlight the interactions between drug targets and disease markers [76], the PPI network was visualized using Cytoscape software version 3.8.0. The CytoHubba plug-in in the software provides 11 topology analysis methods, including degree, which ranks nodes by their attributes in the network, with proteins with high degree tending to be more critical.

#### 4.1.3. GO and KEGG Pathway Enrichment

Gene Ontology (GO) and Kyoto Encyclopedia of Genes and Genomes (KEGG) pathway enrichment analyses were executed on the candidate targets using DAVID 6.8 [77].

*p* values < 0.01 and count value < 0.05 were taken as the thresholds to sort the downloaded results. GO and KEGG are databases of gene-related functions stored based on different classification ideas. Specifically, GO is a database established by the Gene Ontology Consortium, aiming to establish a semantic vocabulary standard that is applicable to various species, limits and describes the functions of genes and proteins, and can be updated as research progresses. GO annotation is divided into MF, BP, and CC, by which the function of a gene is defined and described in multiple ways. KEGG enrichment analysis reflects the metabolic pathways, signal transduction pathways, and disease-related pathways in which genes are involved, revealing the synergistic and regulatory relationships of genes in the overall biochemical reactions and physiological processes of cells, and contributing to the understanding of the role of genes in the development of diseases and the mechanism of drug action. Taken together, GO enrichment focuses more on the categorization and description of gene functions, while KEGG enrichment focuses more on the pathway-level interactions and regulation of genes in biological systems. The combination of the two can assist in more comprehensively and deeply understanding the functions of genes as well as their roles in biological systems.

### 4.2. Experimental Verification

#### 4.2.1. Ethics Statement

The experimental protocol obtained approval from IACUC (Institutional Animal Care and Use Committee of Changchun Normal University, Changchun, China). All experimental procedures followed international standards on the ethical treatment of animals. The experiment adopted the least number of animals to minimize pain.

#### 4.2.2. Reagents and Antibody

Tectoridin was purchased from Shanghai yuanye Bio-Technology Co., Ltd. (Shanghai, China, Cat NO. 611-40-5) with a purity of 98.68%. TritonX-100, Tris, glycine, sodium dodecyl sulfate (SDS), Edaravone and dithiothreitol (DTT) were provided by Sigma-Aldrich, located in St. Louis, MO, USA. Madopar came from (Shanghai Roche Led, Shanghai, China). Primary antibodies against β-actin, Akt, p-Akt (Ser473), Bcl-2, Bax, mTOR, p-mTOR, TLR4, MyD88, NF-κB, Nrf2 and HO-1, horseradish peroxidase (HRP)-conjugated anti-rabbit, and HRP-conjugated rat antibodies were provided by Abcam (Cambridge, UK). All other chemicals were of analytical grade with high purity and were provided by Shanghai Chemical Reagent.

#### 4.2.3. Animals and Treatment

ICR male rats (body weight 25–30 g) were provided by the Shanghai Experimental Animal Center, Chinese Academy of Sciences. We divided 48 adult rats into 6 groups (*n* = 14) in a random manner: the sham group, the model group, the tectoridin at 5 mg/kg, 10 mg/kg, 20 mg/kg group, and the edaravone group (10 mg/kg). Rats received intragastric administration once per day. After 2 weeks of pre-surgery treatment, I/R induced the stroke in the rat following previous description, and some modifications were made [78]. In brief, we inserted a nylon filament (length: 5 cm; diameter: 0.24–0.28 mm) into the middle cerebral artery and made it stay there for two hours. Sham-operated rats received the same surgical procedure as those in the I/R group, except for the middle cerebral artery occlusion. Further, 10 min after the ischemia, the rats were intraperitoneally injected with tectoridin or the same amount of normal saline (NS), and, 2 h later, we carefully took out the nylon filament to make sure that blood could return to the ischemic artery. Then, we sutured the ischemic artery for establishing the reperfusion. After the end of the I/R, we sacrificed the rats. The completion of the behavioral tests was followed by the removal of rats’ brains. Thus, 5 of them were for TTC, 3 for histopathology, and 3 for Western blot analysis.

#### 4.2.4. Neurological Deficit Evaluation

Longa’s neurological severity scale was adopted for evaluating animals’ neurological deficits one day after the sham or I/R surgery, based on previous description [79]. Neurological deficit score involved a 5-point scale: 0, no neurological deficits: 1: incapability of fully stretching the contralateral body and the forelimbs; 2: circling to the contralateral side of surgery; 3: falling to the contralateral side of surgery; 4: unable to spontaneously walk, with reduced consciousness.

#### 4.2.5. TTC Staining and the Infarct Volume Measurement

After the evaluation of the neurological deficits, the rats underwent deep anesthesia for sacrifice. Furthermore, 5 rats were selected from each group in a random manner to receive TTC staining. We carefully took out rats’ brains and cut them into 6 coronal sections (thickness: 2.0 mm). The sections underwent half an hour of 2% TTC staining in NS, followed by one night of fixation in 4% paraformaldehyde solution. The infarcted brain was white, but the non-infarcted region appeared to be pink. Image Pro-Plus 5.1 software assisted in the photographing and measurement of these sections. To eliminate the impact brought about by brain edema, edema in infarct area was corrected, as per previous description [80].

#### 4.2.6. HE Staining

A crown zone (2–6 mm after optico chiasm, hippocampus included) of brain tissues was taken in the embedding box, followed by being conventionally dehydrated, paraffin-embedded, and sliced into 4 μm thick slices. Then, regular HE staining was conducted. Optical microscope (OlympusBX51, Tokyo, Japan) was employed for the observation of brain tissue lesions.

#### 4.2.7. Western Blot Analysis

After neurological deficit evaluation and TTC staining, remaining rats were subjected to Western blot analysis on their brains. The samples underwent the treatment of a lysis buffer (50 mM Tris–HCl (pH 8.0), 150 mM NaCl, 1% Triton X-100, 0.5% sodium deoxycholate, and 0.1% SDS) that contained a cocktail of protease inhibitors (2 µg/mL aprotinin, 1 mM phenylmethyl sulfonyl fluoride, and 10 µg/mL leupeptin). The membranes received one night of incubation of primary antibodies against the Bax, Bcl-2, Caspase-3, p-Akt, Akt, mTOR, p-mTOR, TLR4, MyD88, NF-κB, and β-actin proteins (1:1000) at 4 °C. Washing of blots was completed, followed by 45 min of reaction with peroxidase-conjugated secondary antibodies. The enhanced chemiluminescence (ECL) detection system served to determine the protein concentrations. The Quantity One 4.6.2 software (Bio-Rad Laboratories, Hercules, CA, USA) was adopted for the measurement, quantification, and normalization of the staining intensities of protein bands against the staining of β-actin.

#### 4.2.8. Statistics

All data were in the form of the means ± SD from no less than 3 independent experiments. Student’s t test or one-way analysis of variance (ANOVA) served for a comparison between two groups, and Dunnett’s post hoc test served for a comparison among different groups, under the assistance of Graph Pad Prism 6.0 (Graph Pad Software). *p*-value < 0.05 denoted statistical difference.

## 5. Conclusions

In conclusion, this research marks an inaugural detailed examination of tectoridin’s mechanism in treating IS. Network analysis identified 38 intersecting targets between ischemic stroke and tectoridin. Subsequent analysis through GO functional annotation and KEGG pathway enrichment, supplemented by PPI network analysis and cytoHubba plug-ins, pinpointed twenty-six relevant signaling pathways, including those related to cardiovascular diseases, neurodegenerative diseases, and so on. Network analysis predicted that tectoridin could attenuate brain damage after stroke by modulating the signaling pathways associated with redox, inflammation, and autophagy. The experimental results demonstrated an improvement in neurological function in rats treated with tectoridin, along with a significant reduction in cerebral infarction volume. The neuroprotective benefits of tectoridin stem, in part, from its antioxidant, autophagic, and inflammatory capabilities, which include the up-regulation of Nrf2/HO-1 protein expression, reduction of the TLR4/MYD88/NF-κB inflammatory pathway, and inhibition of the PI3K/Akt/mTOR pathway, contributing to its anti-apoptotic effects. These findings offer fresh perspectives on the molecular mechanisms of tectoridin and provide valuable references for the development of novel anti-IS medications. Further clinical investigation into tectoridin as a promising therapeutic agent in patients with ischemic stroke is warranted.

## Data Availability

The data that support the findings of this study are available within the article.

## Supplementary Materials

The following supporting information can be downloaded at: https://www.mdpi.com/article/10.3390/ijms26041402/s1.
